# Modern Surgery

**Published:** 1852-02

**Authors:** 


MODERN SURGERY.

Prof. Mütter delivered a lecture, a short time since, "on the present position in Europe of some of the most important points of modern surgery," in which he makes the following statement respecting the use of anesthetics:

"In England, Scotland, and Ireland, and on every portion of the continent of Europe, it would appear, that no surgeon of any grade, high or low, pretends to practice his profession without the constant use of some anesthetic agent. When I asked my distinguished friends in London and Paris, if they employed the measure with the same degree of confidence as at first, they seemed surprised at the question, and unhesitatingly declared, that no surgeon would presume to perform a serious operation without first bringing his patient into a state of anesthesia, provided always, there was nothing present to contraindicate the production of this condition. While there exists some difference of opinion as to the best agent to be used, there is none upon the great point of the value of the measure in the practice of surgery. The agent usually employed is *chloroform*, a fluid first used to any extent by Dr. Simpson, of Edinburgh; and it would appear that very few cases of serious consequences resulting from its employment, have been met with either in public or private practice."

Of the use of the microscope in the diagnosis of surgical diseases, he thus speaks:

"*The Microscope.*—The next point of interest to which my attention was directed, was the influence of microscopic observations in surgery. In *Anatomy* and *Physiology* so much has been gained by the use of the microscope, that I was led to hope that our department had also been enriched through the agency of this most wonderful instrument—nor was I disappointed. In the study of *malignant growths*, much has been discovered both novel and practically useful. Again, in the examination of *secretions*, the microscope has proved of great value. For example, we are able to decide in cases of deep ulcer over a bone, by examining the pus, whether or not the bone is diseased. The presence of pus in the sputa can only be detected by the microscope. It is also of great value in the diagnosis of certain diseases of the *kidney and bladder*. Without it, Mr. Quekett could never have made the wonderful discovery that a *urinary calculus*, a solid stone, is in *reality organized;* and by its use alone can we ever arrive at a correct classification of *Tumors*. The application of the microscope in surgical pathology, however, is still almost an untrodden field, and he who now determines to enter upon its cultivation, will most assuredly reap a rich harvest of renown for himself, and confer lasting benefits upon humanity and science."

Prof. Mütter found that in Europe there were still adherents to the old system of dressing wounds with charpie and poultices, and that in France the common practice was still to attempt by such methods the cure of extensive wounds by the *second intention* of Hunter; but that in England, as in our own country, the practice is to secure, in most cases, the immediate union of the wound by *cold water* and the lightest dressings.

